# The potential impacts of circadian rhythm disturbances on male fertility

**DOI:** 10.3389/fendo.2022.1001316

**Published:** 2022-10-06

**Authors:** Tao Li, Yunjin Bai, Yiting Jiang, Kehua Jiang, Ye Tian, Jiang Gu, Fa Sun

**Affiliations:** ^1^ Department of Urology, Affiliated Hospital of Guizhou Medical University, Guiyang, China; ^2^ Department of Urology and Institute of Urology, West China Hospital, Sichuan University, Chengdu, China; ^3^ Department of Otorhinolaryngology, The Ninth People’s Hospital of Chongqing, Chongqing, China; ^4^ Department of Urology, Guizhou Provincial People’s Hospital, Guiyang, China

**Keywords:** circadian rhythm, disturbance, male fertility, infertility, sperm quality

## Abstract

A circadian rhythm is an internalized timing system that synchronizes the cellular, behavioral, and physiological processes of organisms to the Earth’s rotation. Because all physiological activities occur at a specific time, circadian rhythm disturbances can lead to various pathological disorders and diseases. Growing evidence has shown that the circadian clock is tightly connected to male fertility, and circadian perturbations contribute to infertility. The night shiftwork, insufficient sleep, and poor sleep quality are common causes of circadian disturbances, and many studies have reported that they impair sperm quality and increase the risk of male infertility. However, research on the impacts of light, body temperature, and circadian/circannual rhythms is relatively lacking, although some correlations have been demonstrated. Moreover, as the index of sperm quality was diverse and study designs were non-uniform, the conclusions were temporarily inconsistent and underlying mechanisms remain unclear. A better understanding of whether and how circadian disturbances regulate male fertility will be meaningful, as more scientific work schedules and rational lifestyles might help improve infertility.

## 1 Background

As the most predictable environmental change in our planet, day/night alteration is accompanied by oscillations in environmental temperature, humidity, and food availability (1). When the sun rises in the morning, diurnal animals awaken from sleep and plants are exposed to the first light of a new day; thus, all organisms have evolved a universally internal rhythmic timing system to adapt to the Earth’s rotation (2–4). This endogenous system is called circadian rhythm, which is a precise 24-h oscillating cycle to guarantee optimal performance for biochemical functions and physiological processes (1, 5), including sleeping habits, body temperature, feeding behavior, hormone secretion, diet (6, 7) and homeostasis (7–9). A recent paper published in *Nature* also showed that time-restricted feeding (TRF) delayed aging and extended lifespan through circadian-regulated autophagy (10).

Before artificial light was invented, humans modified their behavior to match the natural alteration of day/night (3). Although advancements in modern science and technology have greatly changed our daily living habits and improved human health, they are also accompanied by increased diseases (11). Nowadays, elevated social pressures, overloaded work schedules, and personal habits have caused individuals extensively living with artificial lights or luminescent screens (11) that one-fifth terrain in the planet and four-fifth of the world population (99% individuals for Europe and USA) are exposed to light-populated skies or receive misdirected and obtrusive artificial/outdoor light (12). Meanwhile, prolonged night shiftwork, circadian sleep disorders, jet lag, and distance travel across multiple time zones have become more common (11). All these habits inevitably change the daily wake/sleep cycle and gradually lead to circadian misalignment (11), which finally disturb homeostasis, induce oxidative stress, promote an inflammatory response, and accelerate the coagulation process (7–9, 13). These populations are also more vulnerable to hypertension, diabetes mellitus (DM), hyperlipidemia, obesity, atherosclerosis, and other diseases (7, 11, 14, 15).

Fertility is a fundamental process in animal reproduction and is affected by environmental cues such as light, temperature, rainfall, and food availability (5, 16–19); as such, most species reproduce in times with mild weather and optimal food availability (5, 20). For example, many animals evolve a precise seasonal fertility between spring and early summer; others with longer gestations mate in late summer and autumn, which leads to pregnancy in winter and parturition in spring (21–23). Although humans are not seasonal breeders, their sexual activity (24, 25) and reproductive function are still influenced by the peripheral environment (24, 26), although relatively less (27, 28). Infertility is defined as the inability to successfully spontaneously conceive for at least 2 years of unprotected sexual intercourse and is influenced by occupational and lifestyle factors such as smoking, obesity, alcohol consumption, psychological stress, and lack of exercise (29, 30). Currently, infertility affects nearly 15–20% of all couples worldwide (24, 30) and contributes to various psychological, medical, and financial consequences (30).

In fact, numerous studies have shown that elevated night shiftwork, overloaded work schedule, poor sleep quality, and popularization of mobile phones disrupted the circadian rhythm (5, 27, 28, 31, 32) to impair female fertility by affecting the menstrual cycle and altering hormone secretion, which finally increases the incidence of preterm birth, spontaneous abortion, and membrane rupture and reduces breastfeeding success (1, 23). However, males are estimated to be solely responsible for 20–30% cases of infertility (30, 33), while other studies have even indicated a rate of 50% (34). Nevertheless, whether disturbed circadian rhythms contribute to male infertility, like deteriorating female reproduction, is controversial, and whether a normal or healthy circadian rhythm improves male fertility is more interesting. Herein, we review and discuss the potential relationship between the circadian rhythm disturbances and male fertility.

## 2 Organization and molecular mechanism of the circadian clock system

Circadian rhythm was first described by Konopka in 1971, who cloned the mutant clock gene of *Period* in *Drosophila*; the veil of the circadian clock was subsequently disclosed (1, 35). Recently, the 2017 Nobel Prize in Physiology or Medicine was awarded to Jeffrey C. Hall, Michael W. Young, and Michael Rosbash to reward their huge achievements in exploring and clarifying the regulatory mechanisms in the circadian clock.

**Figure 1:**
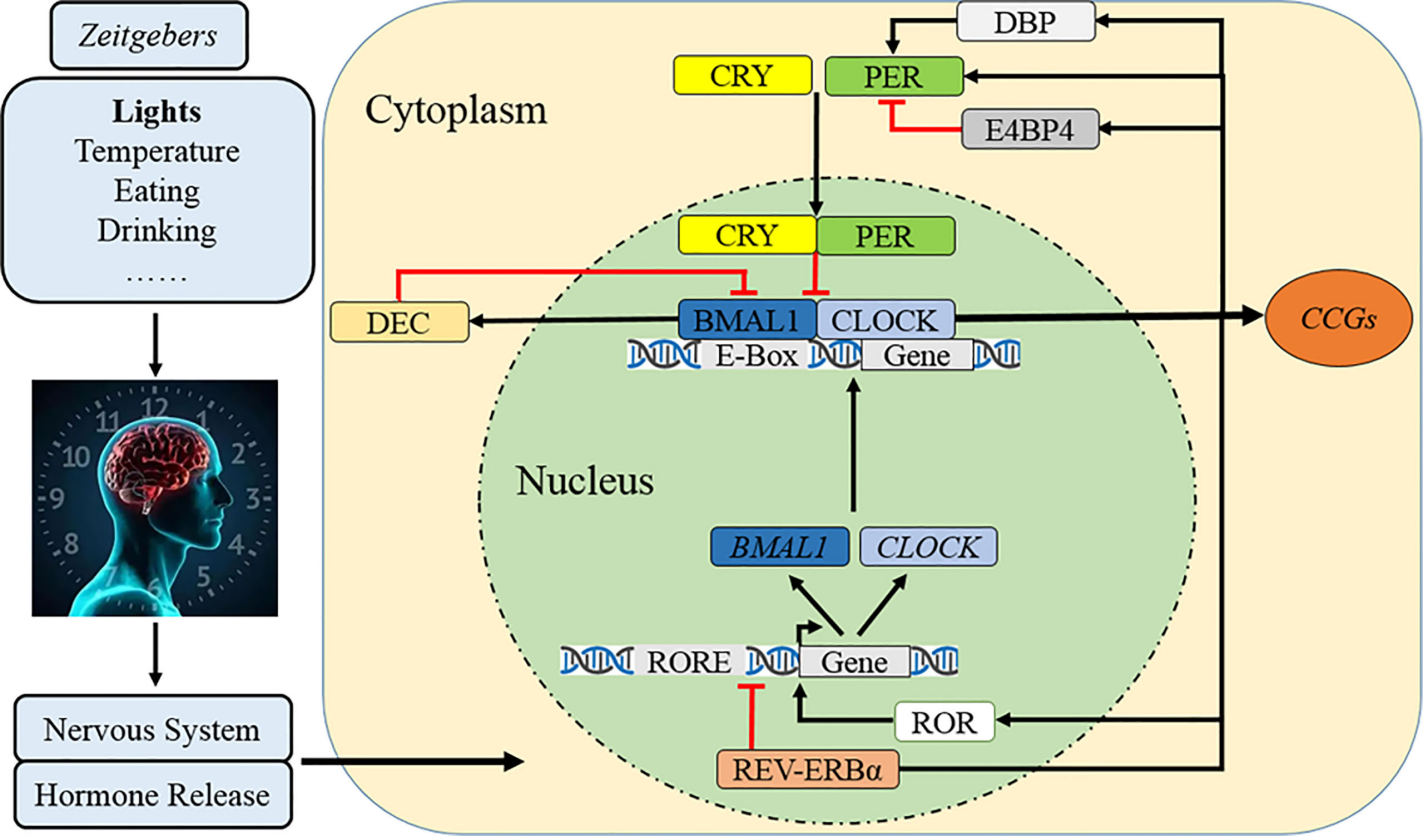
Molecular mechanism of the circadian clock. After cues from Zeitgebers are transmitted to SCN as electrical signals, the circadian clock rhythm is orchestrated by a set of genes and proteins which forms interlocked positive and negative feedback loops. Specifically, CLOCK and BMAL1 constitute the core positive heterodimer complex (CLOCK/BMAL1) to activity rhythmic transcription of *PER1/2/3*, *CRY1/2*, and relevant clock controlled genes (*CCGs*). The repressors of PER and CRY accumulate and form a negative feedback loop in cytoplasm during the day and then translocate back to the nucleus to inhibit the level and activity of CLOCK/BMAL1. The complex of PER/CRY is also subsequently disassembled or resolved, while a new circadian cycle is also followed by another 24–h. Meanwhile, REV-ERBα reduces *BAML1* transcription and RORα promotes it to manipulate the CLOCK/BMAL1 complex. The third loop involves that DBP induces *PER* transcription and lengthens the rhythmic period but E4BP4 exerts opposite effects, while DEC inhibits CLOCK/BMAL1-induced *PER1* transcription.

The circadian clock system consists of three basic elements: the input pathway, the main oscillator, and the output pathway. The input pathway is a passage that modulates the non-24-h central circadian pacemaker to 24-h and adapts the endogenous timing phase to local environment (1). As the main *Zeitgeber* that synchronizes organisms’ circadian clock (9, 36, 37), environmental light is perceived by melanopsin-containing retinal ganglion cells and is transmitted to a central oscillator *via* the retinohypothalamic tract as electrical signal (38) (**Figure 1**). Other factors, such as environmental temperature, feeding behavior, physical exercise, and social interactions, can also be perceived (38). The master oscillator of the circadian clock for mammals, which is composed of a set of circadian clock genes and coded proteins, is located in the suprachiasmatic nucleus (SCN) of the anterior hypothalamus which acts by manipulating the daily endogenous rhythms of physiological and behavioral processes (9). The human SCN comprises approximately 50,000 neurons, including diverse cellular components and numerous neurotransmitters and peptides (39, 40). In addition to the main oscillator, the circadian clock system also exists in almost every peripheral tissue and organ (1, 3, 41). For example, when feeding behavior occurs beyond regular eating time, the circadian timing system in SCN is constant, but rhythms in peripheral organs such as stomach, liver, and pancreas are altered and disturbed, which finally contributes to desynchronization between the internal and peripheral circadian clocks (38). Finally, the output pathway transfers central circadian information to peripheral effector organs (1). The output neural pathways are diverse and include the paraventricular nucleus of the thalamus, subparaventricular zone, medial preoptic area, and dorsomedial nucleus of the hypothalamus (1, 42–46). Hormones are also regulated by the SCN rhythm and are regularly secreted during the day/night cycle (1, 47, 48).

At the molecular level, the circadian clock system is orchestrated by a set of genes and proteins that form interlocking positive and negative feedback loops of transcription and translation (11, 49) (**Figure 1**). In the nucleus, the circadian locomotor output cycle kaput (CLOCK) and brain and muscle ARNT-like protein 1 (BMAL1) constitute the core positive transcription factor (CLOCK/BMAL1) (1, 3, 11, 49). This heterodimer complex binds to a specific E-box (5′-CACGTG-3′) sequence on the promoters of *Period* (*PER1*, *PER2*, and *PER3*), *Cryptochrome* (*CRY1* and *CRY2*), and relevant *clock-controlled genes* (*CCGs*), which finally activate their rhythmic transcription (1, 3, 11, 49). The PER and CRY accumulate and dimerize in the cytoplasm during the day and then translocate back to the nucleus with a timed delay to inhibit the activity of CLOCK/BMAL1 and downregulate the transcriptional activation of downstream circadian genes, forming the core negative feedback loop (1, 3, 7). The repressor complex of PER/CRY is also subsequently disassembled or resolved, while a new circadian cycle occurs in the subsequent 24–h (1, 3, 7). The secondary negative regulatory arm involves the nuclear receptors of orphan nuclear receptor (REV-ERBα/β) reducing *BAML1* transcription and retinoic acid-related orphan receptor alpha (RORα/β) promoting it (7, 50, 51). Another CLOCK/BMAL1 negative loop comprises D-site albumin promoter-binding protein (DBP) and E4 promoter-binding protein 4 (E4BP4) which compete binding to the *PER* promoter of the D-box element (52–54); the upregulated DBP cooperatively induces *PER* transcription and lengthens the rhythmic period but E4BP4 exerts opposite effects (52, 54), while elevated differentiated embryo chondrocyte (DEC) inhibits CLOCK/BMAL1-induced *PER1* transcription by direct protein–protein interactions with BMAL1 and/or competition for E-box elements (55, 56).

## 3 Light exposure and male fertility

**Figure 2:**
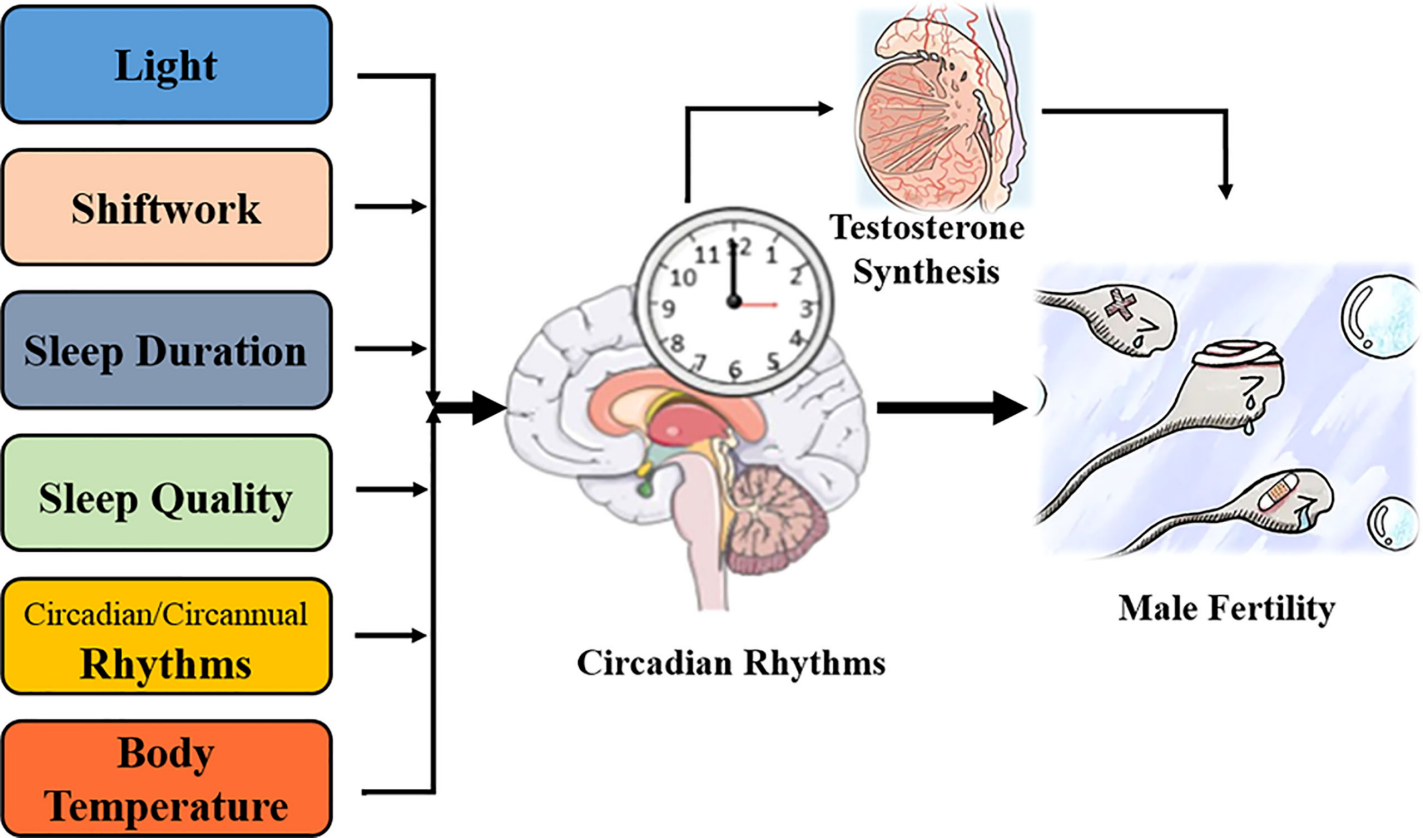
Involvement of circadian rhythms with male fertility.

Light is a fundamental *Zeitgeber* that synchronizes endogenous circadian rhythms with external environments (57). Nowadays, millions of televisions, computers, and smartphones are operated every day (17), and the time spent on these devices peaks in the evening and shortly prior to sleep onset (17). “2011 Sleep in America” also reported that more than 90% individuals used digital screens within 1–h before sleep (17). Thus, the eyes are increasingly exposed to electronic screens or artificial light (17), which contributes to various diseases, including male infertility (**Figure 2**).

A retrospective study recently investigated the impact of digital screen on sperm quality in 116 men (17). Tablet or smartphone usage in the evening or after bedtime was associated with decreased total motile sperm number (−0.173, p < 0.05), sperm progressive motility (−0.322, −0.299, p < 0.05), sperm concentration (−0.169, p < 0.05), and motility (−0.392, −0.369, p < 0.05) while increasing the percentage of immotile sperm (0.382, 0.344, p < 0.05) (17). The authors hypothesized that artificial light chronically impaired the wake/sleep cycle and disturbed the circadian rhythm, which finally deteriorated male fertility by suppressing melatonin levels (17). However, the exact mechanism has not been explored and other artificial illuminations were not analyzed.

Ogo et al. divided pregnant rats into groups of constant light–light (LL) or light–dark (LD) cycles during the gestation period, but all offspring were housed under a normal LD photoperiod until adulthood (57). Offspring in the constant LL group showed a significantly increased number of abnormal sperm heads and decreased normal sperm number (p = 0.0001). The testosterone level, seminiferous tubule diameter, Sertoli cell number, and sperm count in the epididymis were also decreased (57). Moreover, LL reduced glutathione reductase (GR) levels in the epididymis but increased the enzymatic activity of glutathione S-transferase (GST) and glutathione peroxidase (GPx) in the testis (57). These findings suggest that extended light exposure leads to male infertility by decreasing testosterone levels and increasing oxidative stress (57). Moustafa et al. also placed male rats under prolonged light (20-h light and 4-h dark) and darkness (4-h light and 20-h dark) for 12 weeks (57). The prolonged light increased sperm count and motility, while extended darkness reduced the incidence of sperm abnormalities (57). Meanwhile, both extended light and darkness altered hormone secretion and reduced estradiol levels, while increasing FSH, LH, testosterone, and prolactin levels (57). Furthermore, prolonged light exposure increased the expression of *Per1/2* and decreased *Bmal1*, while darkness exposure upregulated *Per1/2*, *Clock*, and *Rer-erab* (57). These results proved that abnormal light exposure disturbed testosterone secretion and the testicular circadian rhythm (57).

Similar to impaired female reproduction, abnormal light exposure may also lead to male infertility (17, 28, 57). However, current results on sperm parameters, hormone levels, and antioxidant indicators are diverse and controversial, while the specific mechanisms of how altered light exposure contributes to male infertility remain to be explored.

## 4 Shiftwork and male fertility

Sleep is a repetitive behavior that synchronizes with daily circadian rhythms, and a regular wake/sleep cycle is essential for human health (29). Shiftwork refers to working beyond the traditional daytime (08:00 to 16:00), which covers rotating or non-rotating (fixed) shift models (29, 58). The European Working Condition Survey in 2005 estimated that more than 17% of staff in the European Union performed shiftwork (29). Several studies have shown that shiftwork disturbed the wake/sleep cycle and impaired circadian rhythms, which contributed to cardiovascular disorders, metabolic diseases, and male infertility (29, 59) (**Figure 2**).

Demirkol et al. found that shift workers (n = 104) had a higher incidence of oligozoospermia (p = 0.006) and lower mean normal morphology (p = 0.036) than non-shift workers (n = 116), while shiftwork (OR = 2.11, 95% CI: 1.03 to 4.34) was independently related to oligozoospermia in multivariate analysis (29). Kohn et al. claimed that shift workers had lower sperm density (p = 0.012), total motile count (p = 0.019), and testosterone levels (p = 0.026) than non-shift workers, whereas no difference was observed in sperm volume and motility or FSH and LH levels (60). El-Helaly et al. explored the impact of occupational exposure on male fertility and found that shiftwork significantly increased the risk of infertility (OR = 3.60, 95% CI: 1.12 to 11.57) (61), which was consistent with findings of Irgens et al. who reported reduced sperm quality among shift workers (OR = 1.46, 95% CI: 0.89 to 2.40) (61). Sheiner et al. also revealed that male infertility was significantly associated with shiftwork (OR = 3.12, 95% CI: 1.19 to 8.13) (62). However, the mechanism by which shiftwork impairs male fertility requires further analysis.

Furthermore, a cross-sectional study with 1,346 men found that rotating shift workers (RSW) had significantly lower total sperm counts (median: 147.3 × 10^6^, interquartile range (IQR): 80.7 × 10^6^ to 255.3 × 10^6^) than day workers (median: 176 × 10^6^, IQR: 101.9 × 10^6^ to 281.2 × 10^6^) (p = 0.034) (63). More RSW (42.4%) revealed lower total sperm count (≤120 × 10^6^) than day workers (30.5%) (p = 0.005); this remained true even after controlling for age, income, smoking, alcohol consumption, BMI, educational level, and abstinence period (OR = 1.60, 95% CI: 1.10 to 2.32, p = 0.014) (63). For permanent shift workers (PSW), LH was the only significantly different parameter (p = 0.044) when compared with day workers (63). In consideration of these differences between the two shift working types, the authors suggested that PSW might align their wake/sleep cycle on workdays with their schedule on days off, which finally decreased circadian desynchrony (63). However, this hypothesis remains unexplored. In addition, a study using a mouse model exposed to photoperiod shifting (light on at 20:00 and off at 08:00) for 28 days showed that the mean total sperm count was significantly decreased by inducing apoptosis in seminiferous tubules, while sperm parameters recovered when light shifting was altered to a constant normal photoperiod for another 35 days (63). However, whether such recovery of sperm quality could be observed in shift workers requires more evidence.

In contrast, Tuntiseranee et al. (64) and Bisanti et al. (65) reported that night shiftwork was not related to male infertility, while another study with 456 males also claimed that shiftwork or night work did not alter sperm quality (66). Considering this discrepancy, further studies with large prospective populations are necessary to verify the impact of shiftwork on male fertility before providing suggestions to shiftwork schedules.

## 5 Sleep duration and male fertility

Similar to proper diet and exercise, normal sleep duration is important for human health (67). The National Sleep Foundation has suggested a sleep period of 7–9 h for people aged 18–64 years and 7–8 h for those over 65 years (68, 69). However, on overloaded work schedule and environmental light/noise pollution, the National Health Interview Survey found that 30% workers (nearly 40.6 million) sleep for <6 h/night in the US (70). Longitudinal studies have claimed that insufficient sleep promoted the risks of mortality, hypertension, T2DM, obesity, and myocardial infraction (69), while others reported a U-shaped association that sleeping for 7–9 h/night had the least risk of deteriorating human health (69, 71). The effect of sleep duration on male fertility has also been explored (69) (**Figure 2**).

Shi et al. enrolled 328 males and found that the sperm concentration remained constant when sleeping for 4.7–8.0 h/night; however, it remarkably dropped when sleeping for <4.7 h/night and noticeably increased when sleeping for >8 h/night (31). Liu et al. observed that sperm count, motility, and survival rates were significantly decreased in participants sleeping for <6 h/night when compared with average (7–8 h/night) or long (>9 h/night) durations (5, 20). Demirkol et al. also reported a positive correlation between sleep duration and sperm concentration in 104 shift workers (29). These results suggest that insufficient sleep significantly impairs sperm quality.

In contrast, an inverse U-shaped relationship between sleep duration and male fertility was observed (72). The Pregnancy Online Study (PRESTO) with 1,176 couples revealed that the fecundability ratio (FRs) for 8 h/night was significantly higher than that for <6 h/night (FR: 0.62, 95% CI: 0.45–0.87), 7 h/night (FR: 0.97, 95% CI: 0.81–1.17), and ≥9 h/night (FR: 0.73, 95% CI: 0.46–1.15), indicating 8 h/night as the most suitable sleep duration for male fertility (73). The Male Reproductive Health in Chongqing College Students (MARHCS) study, which enrolled 796 individuals in 2013, showed that the highest sperm parameters were found for sleeping for 7.0–7.5 h/night, while both shorter and longer sleep durations were correlated with a declined sperm index (p = 0.001 and 0.002, respectively) in a dose-response manner (72). Sleeping for ≤6.5 h/night was related to a 4.6% (95% CI: -10.5 to 22.3) reduction in sperm volume and a 25.7% (95% CI: -1.2 to 60.1) reduction in total sperm number, while sleeping for >9.0 h/night was correlated with 21.5% (95% CI: 9.2 to 32.2) and 39.4% (95% CI: 23.3 to 52.1) reductions, respectively (72). Moreover, sperm parameters were significantly improved when sleep duration was altered to 7.0–7.5 h/night in the following year, which confirmed the sleep–fertility association again (72). However, this damage to sperm quality might not be completely reversible or need more time to recover, as Chen et al. revealed that improved sperm quality was only found in a small (but significant) proportion of patients who changed the sleep duration to a “proper” length (72). High DNA stainability (HDS) in the epididymis is an important index of incompletely differentiated sperms (74). Wang et al. reported that HDS was highest when sleeping for 7.0–7.5 h/night, while those with both shorter and longer sleep durations presented lower HDS (71). A preliminary cross-sectional study based on 92 healthy men also demonstrated an inverse U-shaped relationship between sleep duration and testicular volume, with those sleeping for 9.5 h/night showing the largest volume (75).

These epidemiological studies indicate that sleep duration is strongly associated with male fertility and sperm quality, although the specific positive, negative, or inverse U-shaped association remains to be clarified. Moreover, evidence of long sleep duration impairing male fertility is relatively weak and inaccurate.

From the perspective of animal research, sleep restriction (SR) to 6 h/night (sleeping from 10:00 to 16:00) for 21 days significantly increased immotile spermatozoa and impaired sperm motility, whereas total sperm number and transit time through the epididymis did not change (76). Chen et al. observed that SR for 35 days decreased sperm concentration, viability, and motility, while it increased sperm malformation (77). Alvarenga et al. found that SR and paradoxical sleep deprivation (PSD) resulted in 15% and 50% lower sperm viability, respectively; although sperm concentration was similar, spermatozoa with faster movement were significantly decreased compared to the control group (78). Moreover, Rizk et al. showed that PSD for 5 days significantly increased abnormal sperm morphology but decreased sperm count, viability, and motility (30). Choi et al. reported that SD for 7 days (SD7) significantly reduced sperm motility, whereas SD4 and SD7 partially induced seminiferous tubular atrophy and spermatid retention (32). These results suggest that sleep deficiency impairs sperm viability by disrupting sperm cycle maintenance (78); however, the specific mechanism remains to be investigated.

As sperm quality is mainly regulated by hormonal concentration, numerous studies have focused on the relationship between sleep duration and hormonal levels. The testosterone level is the highest in the morning and lowest during evening, and numerous clinical and basic investigations have shown that sleep deficiency/restriction altered the testosterone concentration by disturbing its secreted cycle (23, 69, 79–81). For example, Chen et al. found that sleeping for 6 h/night for 35 days significantly decreased the testosterone concentration (77) and Alvarenga et al. observed that SD for 96 h significantly inhibited testosterone levels by 45% (78). Choi et al. reported that SD4 and SD7 significantly decreased testosterone release but increased corticosterone production (32). Although the mechanism has not been clarified (77), authors inferred that sleep deficiency increased cortisol and corticosterone levels by activating the hypothalamic–pituitary–adrenal (HPA) axis, the feedback of which inhibited the hypothalamus–pituitary–gonadal (HPG) axis to decrease testosterone secretion (30, 32). In contrast, a preliminary cross-sectional study based on 92 healthy men found that insufficient sleep did not alter total/free testosterone levels (75), while the MARHCS trial found that sleep duration had no impact on reproductive hormones (72). Siervo et al. even found that sleep deficiency significantly increased the testosterone concentration in plasma and intratesticle (76, 82). Some authors thus thought that the altered hormones might not be responsible for decreased sperm quality or infertility (72). As sleep deficiency inhibits testosterone secretion during the second half of a biological night (69, 81), others have suggested that different types and definitions of sleep deficiency may contribute to the controversial interaction between sleep duration and male fertility (69).

Sleep deficiency also damages testes by inducing oxidative stress (76, 82). Akindele et al. found that SR for 14 days significantly elevated testicular malondialdehyde and glutathione (GSH) in adult rats (82, 83). Siervo et al. revealed that SR for 21 days sharply increased the tert-butyl hydroperoxide-initiated chemiluminescence (CL) curve of the epididymidis, which suggested an enhanced peroxidative attack by ROS (76, 82). These results infer that sleep deficiency may impair sperm quality by disrupting the balance between oxidative and antioxidant stress (30, 76). Another male Wistar rat model found that SD (sleeping for 4 h/night) impaired functions of the blood–testis and blood–epididymis barriers by increasing its permeability to low/high-molecular-weight tracers and decreasing the expression of tight-junction proteins, androgen, and actin receptors (84). In this model, rat fertility was improved by recovering sleep for 2–3 days, as the percentage of ejaculating males and impregnated females increased (84).

In general, although more studies tend to show that sleep duration plays an essential role in male fertility and sleep deficiency contributes to infertility, some authors still disagree on the aforementioned correlation as the research designs are diverse and the results are inconsistent. Moreover, exploring how sleep duration regulates male fertility is also a huge challenge considering the complex mechanisms of reproductive function. Finally, whether prolonged sleep duration restores sperm quality remains to be proved.

## 6 Sleep quality and male fertility

Growing evidence has suggested that sleep quality had a significant impact on human health, while poor sleep [defined as difficulties in falling asleep and lying awake at night (5, 85)] increased the risk of hypertension, T2DM, cardiovascular disease, depression, cancer, and male infertility (85) (**Figure 2**).

In 2013, Jensen et al. explored the association between sleep disturbance (based on the Karolinska Sleep Questionnaire) and semen quality in 953 young Danish men (16, 85). Males with both lower and higher sleep scores had significantly decreased total sperm count and concentration, percentage of motile spermatozoa and morphologically normal spermatozoa, and testis size than the control group (sleep score: 11 to 20) (16, 85). Those with a sleep score >50 (poor sleep) had a 29% reduction (95% CI: 2 to 48) in sperm concentration, 25% decline (95% CI: -4 to 46) in total sperm count, 0.9% lower (95% CI: -3.1 to 4.9) motile spermatozoa, and 1.6% fewer (95% CI: 0.3 to 3.0) morphologically normal spermatozoa compared to the control group (16, 85). Kohn et al. also found an inverse U-shaped relationship between sleep quality and sperm quality; the total motile count for moderate sleep quality was 15.4 M sperm/ml greater than individuals without sleep difficulty and 4.72 M sperm/ml greater than those with severe sleep difficulty (p = 0.018) (60).

Some authors have previously evaluated sleep quality using the Pittsburgh Sleep Quality Index (PSQI) global scores. For instance, Chen et al. recruited 842 healthy males which revealed that poor sleep quality (PSQI >5.0) had a lower total sperm count (8.0%, 95% CI: -15% to -0.046%), motility (3.9%, 95% CI: −6.2% to −1.5%), and progressive motility (4.0%, 95% CI: −6.5% to −1.4%) (86). Du et al. conducted a cross-sectional study among 970 patients and demonstrated that increased PSQI scores indicated lower total sperm number (r = −0.160008, p < 0.001), sperm concentration (r = −0.167063, p < 0.001), motility (r = −0.187979, p < 0.001), progressive motility (r = −0.192902, p < 0.001), and normal morphology (r = −0.124511, p < 0.001) (5). Hvidt et al. analyzed the fertility of 140 males and similarly found that sperm quality was reduced when PSQI was increased (p = 0.04) (85). Meanwhile, Viganò et al. observed that sperm motility was decreased for patients lying awake most of the night, whereas sperm volume was lower and concentration was higher for those with difficulty in initiating sleep (87).

Studies have suggested that poor sleep quality might decrease the serum testosterone concentration (86, 88) and impair Sertoli cells in the seminiferous tubules (32, 86). However, Jensen et al. (16), Chen et al. (72), Ruge et al. (89), and Du et al. (5) did not observe any correlation between sleep quality and reproductive hormone levels. In general, although some authors speculated that poor sleep quality (86, 90) or inappropriate sleep habits (5, 38, 91) disturbed the circadian rhythms and damaged male fertility, the results were still inconsistent and the evidence was weak. Further epidemiological and fundamental studies are required to clarify this issue.

## 7 Circadian/circannual rhythms and male fertility

As the two most prominent biological rhythms, day/night alterations (circadian rhythm) and seasonal changes (circannual rhythm) play essential roles in numerous biological functions, including male fertility or sperm quality (24) (**Figure 2**).

The MARHCS dataset, which analyzed 10,362 community populations, found that the sperm DNA fragmentation index (DFI, the most frequent parameter to assess sperm chromatin integrity) decreased from 08:00 to 11:00 (p = 0.335) and then increased after 12:00 (p < 0.001), while the DFI was elevated by 4.2% per hour (95% CI: 1.9 to 6.7, p < 0.001) after 11:00 (92). The Reproductive Medical Center (RMC) dataset with 630 clinical populations showed that sperm DFI decreased before 11:00 (p < 0.01) and each hour (after 07:00) was associated with a 3.8% reduction (92). The rat model proved that sperm DFI varied at different times (p = 0.05); it decreased from 03:00 to 07:00 (p = 0.038) and then increased until 23:00 (p = 0.002) (92). Further cosinor analysis indicated a nadir of DFI at 10:00 (92). Xie et al. analyzed 12,245 sperm samples and found that semen collected before 07:00 had the highest total sperm count and concentration, as well as normal sperm morphology (24). Moreover, Shimomura et al. observed that the total motile sperm count and total sperm count were significantly higher in samples collected in the evening than in the morning (34). This suggests that sperm quality was lower in the morning, while evening-collected semen might be easier to achieve successful intrauterine insemination (34).

The circannual rhythm also significantly influences reproductive behavior, especially in mammals (24). To guarantee the greatest survival chances, many species have adapted to ensure that offspring are born at the most suitable time of the year, when climate and food are the most favorable (24). Although humans are not seasonal breeders, sexual activity (24, 25) and reproductive function still alter with a circannual rhythm (24, 26). Xie et al. analyzed 12,245 semen samples and proved that total sperm count and concentration were higher in spring and lower in summer, while morphologically normal spermatozoids were significantly increased in summer (24). The authors suggested that photoperiod alteration was the most likely cause of circannual variation in sperm quality (24, 93), while temperature variation might be another reason for high hyperpyrexia impaired spermatogenesis (24, 93). Circadian clock genes were also involved, as Akiyama et al. reported that *Bmal1* expression in the hypothalamus and testes was significantly decreased in the transitional season compared to the reproductive and non-reproductive seasons; the *Cry1* level also sharply declined in the transitional season compared to the reproductive season. The testicular morphology and circadian clock genes (*Bmal1* and *Cry1*) revealed circannual alterations (94). However, whether and how circadian clock genes regulate male fertility in circannual rhythms requires further exploration.

## 8 Body temperature and male fertility

Similar to light alteration and wake/sleep cycle, daily oscillation of environmental temperature is another typical *Zeitgeber* to synchronize the internal circadian system; thus, body temperature also presents a 24-h oscillation to adapt to the Earth’s rotation (95). High ambient temperatures can reduce sperm production by destroying the spermatogenic epithelial structure, inducing testicular oxidative stress, and promoting germ cell apoptosis (95–98). Meanwhile, hyperthermia regulates testicular function by altering the daily secretion of reproductive hormones and changing the expression of circadian and testosterone synthesis-related genes (95) (**Figure 2**).

Li et al. exposed male mice to hyperthermia (39°C) from 11:00 to 15:00 (4 h/day) for 35 days (95). This hyperthermia disturbed the rhythm of testosterone secretion, which increased at 11:00 h and decreased at 15:00 h (95). Hyperthermia promoted the transcription of testicular *Star* and *Ar* at 11:00 and enhanced the protein level of CYP11A1 at 23:00 (95). Moreover, heat exposure stimulated testicular *Clock* expression but decreased its protein content at 11:00 and increased the BMAL1 concentration at 23:00 (95). This suggests that high external temperature arrests spermatogenesis by disrupting the rhythms of testosterone secretion and clock genes (95). Sabés-Alsina et al. maintained New Zealand White rabbits under environmental temperatures increasing from 22°C to 31°C (maintained for 3 h) and then gradually declined to 22°C until 09:00 on the following day (99). Hyperthermia significantly decreased the percentage of viable spermatozoa (74.21% *vs*. 80.71%) (p < 0.05) and increased the ratio of acrosomic abnormalities (36.96% *vs*. 22.57%) and tailless spermatozoa (12.83% *vs*. 7.91%) (p < 0.01) (99). However, heat exposure did not alter motility parameters or fertility and prolificacy rates (99). The lack of impairment on fertility and prolificacy may be due to the rapid recovery of reproductive function (99).

In summary, studies on the impact of environmental temperature on sperm quality are diverse and definite; however, whether the oscillation of body temperature influences male fertility and, if so, how it works remains a mystery.

## 9 Clock genes and testosterone synthesis

Although the diverse reproductive hormones are involved in regulating male fertility and facilitating the spermatogenetic process, only testosterone is essential to maintaining spermatogenesis (100). Mainly released by Leydig cells, the serum testosterone concentration presents rhythmic oscillation in adult male mice and human (101–104) which starts to rise at sleep onset (peaks around 8:00) but falls during the day (trough around 20:00) (104, 105). It is reported that circadian misalignment shifts the summit value of the testosterone’s diurnal rhythm to happen soon after waking up (106); however, whether circadian disturbance alters the testosterone concentration is controversial (5, 15, 85, 104, 106–108) and which clock element regulates its secretion is also unknown (103) (**Figure 2**).

Alvarez et al. have found that BMAL1 protein was rhythmically expressed in mouse Leydig cells (103). Since then, most of the core clock genes like *Bmal1*, *Per1/2/3*, *Cry1/2*, *Rev-erbα/β*, *Rorb*, and *Dbp* in Leydig cells were demonstrated to rhythmically oscillate (101–103, 109), except *Clock*, *Rora*, *Ck1δ*, and *Ck1ε* (102). Meanwhile, the steroidogenic-related genes which are responsible for testosterone production in Leydig cells (including *Star*, *Cyp11a1*, *Cyp17a1*, *Hsd3b2*, *Hsd17b3*, *Sf1*, positive-*Nur77*, and negative-*Arr19*) also exhibited 24-h rhythmic expression patterns (101, 102, 109, 110). Specifically, Baburski et al. found the summits of *Star*, *Cyp11a1*, and *Cyp17a1* oscillation occurred approximately at the middle of the light phase, i.e., a few hours before the testosterone release peak (102). These results indicate a crucial role of the circadian clock in testosterone production (101–103, 110). Furthermore, *Bmal1* knockout or inhibition was reported to decrease testosterone secretion by reducing mRNA levels of steroidogenic genes (*Star*, *Cyp11a1*, *Hsd3b2*, *Hsd17b3*, *3β-Hsd*, *Sf1*, and *Nur77*) (101, 103, 111, 112) and Apo (*Apoa1/2* and *Apoc3*) (112). Moreover, *Bmal1* knockdown inhibited testosterone level by inducing apoptosis of Leydig cells (111), and the circadian clock system was involved to the process of bisphenol A (113) and zearalenone (114) reducing testosterone production. In a word, circadian rhythms can regulate testosterone production by various signaling pathways.

## 10 Conclusion

The circadian rhythm is strongly correlated with human health, while growing evidence suggests that circadian disorders contribute to male infertility. With the high incidence of night shiftwork, sleep deficiency, and poor sleep quality in modern life, numerous studies have investigated their influences on fertility and found that they impaired sperm quality and increased the risk of male infertility. Evidence for the impacts from light, body temperature, and circadian/circannual rhythms is relatively weak, although some correlations have been uncovered. However, the current conclusions were inconsistent as the abundant indices of sperm quality and male reproduction, while how the circadian clock genes were involved also remained to be further explored. Nevertheless, a better understanding on the interaction between circadian rhythm disturbance and male fertility will be meaningful, as a more scientific and rational lifestyle and work schedule might help to improve infertility.

## Author contributions

All authors researched data, made substantial contributions to discussion content, and edited the manuscript before submission. TL, YB and YJ wrote the article, JG and FS guided and revised it. All authors contributed to the article and approved the submitted version.

## Funding

This manuscript was funded by the National Nature Science Foundation of China (No. 82060276) and the Science and Technology Department of Guizhou Province [QianKeHeJiChu-ZK(20210)YiBan382].

## Conflict of interest

The authors declare that the research was conducted in the absence of any commercial or financial relationships that could be construed as a potential conflict of interest.

## Publisher’s note

All claims expressed in this article are solely those of the authors and do not necessarily represent those of their affiliated organizations, or those of the publisher, the editors and the reviewers. Any product that may be evaluated in this article, or claim that may be made by its manufacturer, is not guaranteed or endorsed by the publisher.
